# Harnessing AACR Project GENIE to Define the Molecular Features of Desmoplastic Small Round Cell Tumor

**DOI:** 10.3390/cimb48010085

**Published:** 2026-01-15

**Authors:** Sowmya Kolluru, Nicole Horio, Elijah Torbenson, Beau Hsia, Abubakar Tauseef

**Affiliations:** 1 School of Medicine, Creighton University, Omaha, NE 68178, USA; spk88510@creighton.edu (S.K.); nicolehorio@creighton.edu (N.H.);; 2 Creighton University School of Medicine, Phoenix, AZ 85012, USA; 3 Department of Internal Medicine, Creighton University School of Medicine, Omaha, NE 68124, USA

**Keywords:** AACR, desmoplastic small round cell tumor, cancer genomics, genome sequencing

## Abstract

Desmoplastic small round cell tumor (DSRCT) is a rare but aggressive soft tissue sarcoma of the abdomen. With an asymptomatic course and rapid dissemination, DSRCT’s prognosis is poor at diagnosis. This study characterizes the demographic variation and genomic profile of DSRCT to guide studies into diagnosis and treatment. The AACR GENIE database was utilized to identify genetic alterations in DSRCT. Data was queried to identify disease prevalence by different demographic variables. Information was collected on frequency of somatic mutations and copy number alterations, rates of mutation co-occurrence, and mutations seen in primary and metastatic samples. ARID1A, TP53, ATM, TERT, and FGFR4 were the most frequently identified somatic mutations. Copy number alterations seen in DSRCT were commonly homozygous deletions in tumor suppressor genes. Independent of sex, WT1 mutations were most common. Non-White patients saw single occurrences of many mutations but recurrent ones in ANKRD11 and KMT2C. Co-occurrence was found between FGFR4 and EP300. Moreover, primary tumor samples had exclusive mutations in AKAP9, KDM2B, MAGED1, MKI67, PCLO, and TRAF1. Metastatic samples had exclusive mutations in FIP1L1 and NRIP1. Our data highlights mutational variation across demographic cohorts. These patterns are vital to future studies into identifying diagnostic markers or therapeutic targets.

## 1. Introduction

Desmoplastic small round cell tumor (DSRCT) is a rare but aggressive sarcoma primarily affecting adolescents and young adults [1]. Per the 2020 International Classification of Diseases for Oncology, DSRCT is considered a malignant tumor of uncertain differentiation [2]. Though primarily localized to the abdomen, DSRCT can appear in various organ systems, including the lungs, heart, colon, bones, and gonads [3]. With origins in mesenchymal stem cells and progenitor cells, DSRCT is molecularly defined by EWSR1:WT1 gene fusion (t(11,22)(p13;q12)) [4]. With no other known significant risk factors, DSRCT poses a unique diagnostic and treatment challenge as it presents as numerous peritoneal masses, often with one more prominent mass, rather than a single tumor. Moreover, DSRCT remains largely asymptomatic until the tumors become more invasive, causing ascites [5]. As such, these patients present clinically with signs of abdominal distention, pain, and obstruction [5,6]. With its rapid dissemination, sarcomatosis is often diagnosed at advanced stages, evidenced by a 5-year survival rate of between 15 and 30% and a median survival of 17 to 25 months [5,7].

A rare tumor, DSRCT cases have been reported around the world with an annual incidence of between 0.2 and 0.5 cases per million individuals [8]. DSRCT can affect individuals of all age groups, but on average, it presents in individuals between the ages of 20 and 30 [5,9]. DSRCT is more prevalent among males, who account for 85–90% of diagnoses. Still, at younger ages, females are more likely to be diagnosed with this disease [5]. Racial disparities also exist. DSRCT is both more common and associated with higher mortality among the African American population compared to the White population in the United States [10,11].

Diagnosis of DSRCT is essential to mitigate the mortality burden. Diagnosis can be made by core-needle biopsy, diagnostic laparoscopy, or laparotomy [2]. DSRCT presents very similarly on histopathology to other small round cell tumors, such as Ewing Sarcoma and Rhabdomyosarcoma, complicating diagnosis [4]. The unique fusion gene present in this tumor is typically confirmed with fluorescence in situ hybridization or reverse-transcription polymerase chain reaction [2]. Through immunohistochemical staining, markers of mesenchymal, epithelial, and neural crest origin, including desmin, cytokeratin, and S100, may all be detected [5]. Staging of DSRCT remains a challenge. With no specific staging protocol and the disseminated nature of the disease, use of the Union for International Cancer Control (UICC) staging system results in most patients being diagnosed as stage IV [7]. As this cancer often presents with multiple abdominal masses, direct resection alone is typically insufficient [11]. Over 90% of cases require a combination of surgery, alkylating chemotherapy, and radiation [2]. Cytoreductive surgery, an intra-abdominal tumor resection strategy, has been associated with increased survival in patients with this disease [2]. Despite these combination treatment efforts, however, mortality remains high, with many patients succumbing to the disease within 3–5 years [12].

The EWSR1-WT1 fusion protein remains an important alteration present in DSRCT. This protein acts as a transcription factor, binding DNA and modulating oncogenesis [13]. Aside from this alteration, there is minimal overlap in the prevalence of other genetic alterations among different tumor specimens. Another mutation, ARID1A, has also been identified among some specimens [14]. ARID1A, a tumor suppressor, has been associated with DSRCT progression when mutated [14,15]. Still, with much variation in the genetic features of DSRCT outside of its recurring fusion protein, there is a need for improving our understanding of its genetic and molecular profile.

DSRCT remains a significantly understudied but highly aggressive soft tissue sarcoma. With patients being excluded from research studies and the poor survival rates limiting options for study, there is very limited information on management of patients with this diagnosis [1]. Given the insufficiency of current treatment regimens, there is a growing importance for understanding the demographic variables that influence diagnosis and the various somatic mutations, copy number alterations, and other DSRCT-associated genetic alterations to guide development and study of novel therapeutics to which this tumor is susceptible.

## 2. Materials and Methods

De-identified case information and data via the American Association for Cancer Research Project Genomics Evidence Neoplasia Information Exchange database (AACR GENIE) were utilized to build a demographic and genomic profile of DSRCT. In June 2025, cBioPortal (v17.0public) was queried to specifically profile genomics and demographics associated with DSRCT for data analysis. As the study utilizes publicly available and de-identified patient information, this study is exempt from IRB approval.

The AACR GENIE database is a cancer database founded in 2015 that provides information on over 110,000 tumors from over 100,000 patients treated at 20 cancer institutes across the world with the goal of guiding cancer research and care [16,17]. Whole-genome sequencing, whole-exome sequencing, and targeted gene panel information available within the database were assessed. About 80% of samples underwent targeted gene panel sequencing with a coverage of over 500 reads. Whole-exome sequencing was completed for about 15% of samples with a coverage of 150 reads each, and whole-genome sequencing was completed for 5% of samples for a coverage of about 300 reads. Though 65% of the sequenced samples only assessed tumor-specific characteristics, 35% of tumor samples were sequenced alongside a matched normal sample to aid in improved identification of mutations and differentiate inherited and acquired mutations.

In addition to the unique annotation and variant-description techniques each of the international cancer treatment centers utilize in their data collection, AACR GENIE ensures standardization and consistency of submitted data through its own Genome Nexus protocol, which includes using GATK to identify variants and ANNOVAR to annotate and describe them. Because multiple pipelines were in use during the consortium data submission period, software version numbers varied by institution. Though treatment regimen and outcome data were available for some of the cancers in the database, it was unavailable for DSRCT. To describe the genomic profile of DSRCT, targeted gene sequencing, whole-genome sequencing, and whole-exome sequencing were completed.

In this study, we collected information on patients diagnosed with DSRCT. This patient cohort was then subdivided into those with primary cancer and those with metastatic cancer, and Chi-square analysis was completed to compare the frequency of mutations in these two categories. Genomic data, including somatic mutations and copy number alterations, and demographic information, including sex, age, race, and ethnicity, were collected. Though there was variation in the genes highlighted by different institutions, key genes, including TP53, were submitted by most. Genes for which data was missing or of unknown significance were excluded from the study, as were larger structural variants. To calculate gene frequencies, the total number of patients in the group was preferentially used as the denominator, rather than the number of samples analyzed per gene. This ensured that we avoided overestimation of gene frequencies that were only assayed in some of the samples.

Copy number alterations (CNAs) were another major focus of this study. We assessed the frequencies of common homozygous deletions and amplifications seen in the DSRCT cohort. Tumor mutational burden was obtained with the support of the AACR GENIE statistical team to understand the relationship between DSRCT and other variables. Tumor mutational burden, defined as mutations per megabase, was estimated from gene panels by studying synonymous mutations, nonsynonymous mutations, and genetic hotspots [18]. Linear regression was utilized to harmonize calculated tumor mutational burden measurements against the whole-exome sequencing-based tumor mutational burden.

Samples for which data was either missing or of unknown significance were excluded from statistical analysis, which was conducted using R with statistical significance designated as *p* < 0.05. Categorical variables we studied are reported as counts and frequency, and the strength of association between these variables was assessed with Chi-square analysis. For our subgroup analyses, results were not corrected for multiple comparisons due to limited sample size. As such, subgroup analysis is exploratory and should be interpreted as such.

Nonsynonymous somatic mutations with a variant allele frequency ≥5% and a coverage greater than or equal to 100 reads were included. Both synonymous somatic mutations and those of unknown significance were not. The total number of samples profiled per variation was utilized for the determination of both CNAs and structural variant frequencies. Mutations were identified and described from AACR GENIE’s mutation annotation format files. These files serve to highlight various gene mutations, mutation types, origins, and annotations to ensure standardization of genomic data sharing across participating international cancer treatment centers.

## 3. Results

### 3.1. Demographic Data for Desmoplastic Small Round Cell Tumor

This study reports data from 202 samples and 142 patients. There were 117 (82.4%) males and 22 (15.5%) females, with 3 (2.1%) unknown. In our data, non-Hispanic patients accounted for 107 (75.4%) and Hispanic patients accounted for 23 (16.2%), with 9 (6.3%) unknown or unreported. Of the patients included in this study, 12 (8.5%) were Asian, 84 (59.2%) were White, 27 (19%) were Black, 7 (4.9%) were other, and 9 (6.3%) were of unknown ethnicity. Moreover, 118 patients (58.4%) were aged >18 years old, and 84 (41.6%) were ≤18 years old. Data was also collected on whether the tumor was primary or metastatic. While 95 (47%) were primary, 97 (48%) were metastatic and 10 (5.0%) were classified as unknown. Patient demographic data is reported in Table 1.

### 3.2. Frequent Somatic Mutations

Table 2 highlights frequent somatic mutations found in this DSRCT cohort. The most common mutations were identified in *ARID1A* (n = 16; 7.9%), *TP53* (n = 5; 2.5%), *ATM* (n = 7; 3.5%), *TERT* (n = 6; 3.0%), *FGFR4* (n = 5; 2.5%), *EP300* (n = 4; 2.0%), *ALK* (n = 3; 1.5%), *NOTCH1* (n = 4; 2.0%), *KMT2C* (n = 4; 2.0%), *MTOR* (n = 4; 2.0%). According to this data, mutations in *ARID1A* were the most prevalent, with *TP53* and *ATM* mutations also occurring at notable frequencies.

### 3.3. Frequent Copy Number Alterations

Recurrent CNAs were also identified and are reported in Table 3. The most common CNAs were homozygous deletions in tumor suppressor genes, including *CRLF2* (n = 7, 3.6%), *PTEN* (n = 5, 2.6%), and *FAT1* (n = 5, 2.7%). *CDKN2A* (n = 4, 2.1%) and *CDKN2B* (n = 4, 2.1%) deletions were less frequent but still reported. Less prevalent were amplifications, which occurred in genes such as *TMPRSS2* (n = 4; 2.1%) and *ERG* (n = 3, 1.6%), *ICOSLG* (n = 3, 1.7%), *U2AF1* (n = 3, 1.6%), and *ELF3* (n = 3, 2.0%).

### 3.4. Notable Mutations by Sex and Race

The first demographic variable by which we studied our sample was sex. Mutations in *WT1* were the most common, found in 84.80% of men (n = 145) and 68.00% of women (n = 17) with *p* = 0.0489. Mutations in *NSD1* were of the more frequent mutations reported (n = 5, *p* = 0.0142) and were seen in both males and females. Mutations in *KDM2B*, *MAGED1*, *MKI67*, *PCLO*, and *TRAF5* were reported only in males, each with a single occurrence (n = 1). Mutations in *FL1* were found in a single female patient, while RUNX1 mutations were identified in two female patients. Key genetic mutations are stratified by sex and reported in Table 4.

In this cohort, several mutations were observed only once (n = 1) in Non-White patients, including FLI1 (*p* = 8.33 × 10^−3^), KDM2B (*p* = 0.0165), MAGED1 (*p* = 0.0165), MKI67 (*p* = 0.0165), PCLO (*p* = 0.0165), and TRAF5 (*p* = 0.0165). ANKRD11 (n = 3 vs. n = 1; *p* = 0.0344) and KMT2C (n = 4 vs. n = 1; *p* = 0.0411) mutations were also significantly enriched in Non-White patients. The differences in recurrent mutations between White and Non-White patients are highlighted in Table 5.

### 3.5. Co-Occurring and Mutually Exclusive Genetic Alterations

*FGFR4* mutations frequently co-occurred with *EP300* mutations (n = 2/10; *p* = 0.009). Though not statistically significant, *TP53* co-occurred with *MTOR* (n = 1/10, *p* = 0.122), *TERT* co-occurred with *FGFR4* (n = 1/11, *p* = 0.162), and *ARID1A* co-occurred with *ALK* (n = 1/21, *p* = 0.272). Data collected on mutual exclusivity of gene mutations were not statistically significant (*p* > 0.05).

### 3.6. Genomic Alterations Among Primary and Metastatic DSRCT

The overall study cohort comprised 95 primary and 97 metastatic DSRCT cases, and all were included in the comparative genomic analysis. These analysis group sizes (n = 95 vs. n = 97) were of comparable size, minimizing potential bias. Among the 95 primary tumor samples, AKAP9, KDM2B, MAGED1, MKI67, PCLO, and TRAF1 mutations were identified as single-occurrence events and were absent in the metastatic samples. Conversely, FIP1L1 and NRIP1 mutations were each observed once in the metastatic samples and not detected in primary tumors. Although there were genes exclusively identified in primary or metastatic samples as demonstrated above, key characteristics of the mutational landscape, including frequencies of recurrent alterations in genes like ATM, NOTCH1, and FH, showed substantial overlap and no significant differences between the groups.

## 4. Discussion

The goal of this study is to build a robust genomic and demographic profile of a rare but largely aggressive and understudied cancer, DSRCT, using the AACR GENIE database. Our data highlights mutational variation across the different demographic cohorts studied.

According to the patient data gathered from AACR GENIE, DSRCT was most prevalent in males, with 82.4% of the samples being males (n = 117) and 15.5% being females (n = 22). The current literature highlights a similar trend, with one identifying that 85–90% of patients are males and another highlighting a 3.8:1 male/female predominance [5,19]. DSRCT was more prevalent in patients aged >18 than in those aged ≤18 years old, with a 58.4% and 41.6% frequency, respectively. DSRCT is a cancer of young adults and adolescents, affecting these groups at higher rates than the elderly population. In fact, the current literature strengthens this finding, demonstrating a peak incidence between ages 20 and 24 [11]. This trend can be explained by the tumor’s origin in mesenchymal stem cells and primitive cells, which have more potential and ability to differentiate in younger individuals [4,20]. While prior literature demonstrates an increased prevalence of DSRCT among the African American population compared to their White counterparts, our data shows an increased frequency among White (n = 84) and Black patients (n = 27) than their Asian (n = 12) counterparts. The AACR GENIE database providing insight into DSRCT prevalence internationally, rather than localized to the United States, may explain the increased prevalence of the tumor among the White population [10,11]. It is important to note that the conducted subgroup analyses are not corrected for multiple comparisons should be interpreted as exploratory findings.

Numerous frequent somatic mutations have also been identified by this study, including *ARID1A* (n = 16; 7.9%), *TP53* (n = 5; 2.5%), *ATM* (n = 7; 3.5%), *TERT* (n = 6; 3.0%), and *FGFR4* (n = 5; 2.5%). This both aligns with and builds on the prior literature highlighting somatic mutations, including ARID1A, HRAS, TP53, TERT, MSH3, and FGFR4, among others, involved in DSRCT pathogenesis [8,21]. WT1 mutations were most common in both males (84.8%) and females (68.0%). As larger structural variants were excluded from the study, these alterations represent sequence variants, rather than the result of fusion events. The existing literature identifies the EWSR1:WT1 gene fusion as the pathognomonic and defining molecular event in diagnosis of DSRCT [4,22]. Given the critical role of WT1 in DSRCT pathogenesis, characterization of specific WT1 alterations highlights a key gene that can serve as a therapeutic target.

EWSR1:WT1 gene fusion is central to characterizing DSRCT, making it an important consideration for guiding therapeutic strategy. The protein is generated by the fusion of the N-terminus of EWSR1 on chromosome 22 to the C-terminus of WT1 from chromosome 11. As a result, the transcription activation component of EWSR1 fuses with the DNA-binding component of WT1, leading to oncogenesis [23]. EWSR1:WT1 acts as a transcription activator, acting on an array of downstream targets, including MYC, EGFR, CHD1, and MTOR [14]. Activation of these oncogenes can contribute to cancer progression. This fusion gene also has the capacity to activate downstream CCND1, a gene that encodes a key protein involved in cell cycle progression. In fact, CDK4/6 inhibitors aimed at inhibiting CCND1 have been promising in early in vitro and in vivo models of DSRCT [24].

FGFR4 mutations frequently co-occurred with EP300 mutations (n = 2; *p* = 0.009). FGFR4 is a receptor tyrosine kinase involved in cell growth, survival, and metastasis. It is involved in the activation of other proliferation pathways, including PI3K-AKT and JAK/STAT [25]. EP300 is an important tumor suppressor gene involved in the regulation of the cell cycle [26]. The co-occurrence of mutations in these two genes may suggest a synergistic effect on cell cycle dysregulation. Though an interesting finding, it is important to note that our data reports a *p* value that indicates statistical significance despite a minimal n = 2 sample size, which inherently limits statistical robustness and interpretability. Further study in larger cohorts is needed to validate these findings. Still, this finding suggests that further study is needed to characterize the molecular mechanisms of their interactions for the development of potential novel combined therapeutic strategies.

Genetic variation was also evident according to primary or metastatic cancer designation. Among the primary tumor samples, AKAP9, KDM2B, MAGED1, MKI67, PCLO, and TRAF1 mutations were identified. These mutations were all absent in metastatic tumor samples, which presented with FIP1L1 and NRIP1 not observed in the primary tumor samples. Still, certain mutations, including ATM, NOTCH1, and FH, were identified in similar frequencies in both sample groups. While limited literature has shown the association between FIP1L1 and metastases, NRIP1 is involved in the epithelial-to-mesenchymal transition, or EMT, which can lead to tumor migration and invasion [27]. This may explain why NRIP1 is exclusively found in metastatic DSRCT. Our data adds to the current literature that also suggests higher rates of SOX2 and NANOG in metastatic DSRCT compared to primary cancer [13]. It is important to note that the database includes every sample obtained, be it primary or metastatic. As such, multiple samples may have been obtained from the same patient and de-duplication was not performed. Our Chi-squared analysis implies independence, which may be limited by lack of de-duplication. This is an important consideration while interpreting the results. Still, the findings still emphasize how understanding of genetic mutations found in metastatic tumors and primary tumors can help guide patient-specific treatment of DSRCT.

This study helps characterize variation by demographic factors in the presentation of DSRCT and describes the genetic profile of the cancer. This information is vital to guiding future targeted studies into the diagnosis and treatment of the cancer.

## 5. Limitations

This study is not without limitations. Firstly, AACR GENIE is limited to the information compiled and shared by the twenty participating institutions. As such, the study is limited both in sample size and by the sequencing strategies and approaches utilized by different participating treatment centers. With a smaller sample size, certain mutations present as single occurrences, making both the trends and the clinical outcomes of the mutations difficult to identify. Further studies that compile data across databases and from additional international treatment institutions will help elucidate these gaps in knowledge. Additionally, variations in the sensitivity of sequencing utilized by different institutions and the number of reads performed for coverage can influence the obtained data. Different pipeline protocols used by different programs can lead to misrepresentation of diagnostic ambiguity and result in missing data when frequencies are low. AACR GENIE also does not include information on prognosis and response to treatment in DSRCT patients with varying genomic profiles. Insufficient information is available to derive further conclusions on which collection of mutations each patient has, and which would contribute to improved success or worsened outcomes with treatment. Moreover, while multiple mutations were identified, their level of expression and possible epigenetic influences cannot be deciphered. Similarly, we are unable to identify which mutations are drivers of disease, leading to the onset of cancer progression, and which are mutations acquired later in the course of the disease. Though unlikely to significantly affect the findings, AACR GENIE includes both independent and non-independent samples, for which primary and metastatic samples are included for the same patient, but the variation by sample per individual patient cannot be identified. In addition, classification of samples as “primary” or “metastatic” is based on clinical annotations likely reflective of sampling site rather than true biological stage. Given that DSRCT frequently presents with widespread disease at diagnosis, distinction between primary and metastatic lesions is inherently challenging and often speculative [5,6]. Accordingly, interpretation of comparisons between these groups should consider the difficulties in this classification. With DSRCT presenting with immunohistochemical cell markers of various subtypes, including neural crest, epithelial, and mesenchymal, further study is needed to identify how variations in histochemical profiles influence prognosis and treatment planning. In addition, genes that were not assayed on any given gene panel were automatically assigned as wild type. To minimize this effect, we prioritized only the most frequently mutated genes that were included in most of the gene panels. Mutation frequencies were also calculated as a fraction of the total number of patients, rather than number of samples studied by genes to prevent overestimation for genes that were not assayed for every sample. In addition, this study includes numerous subgroup analyses without correction for multiple comparisons, increasing the risk for Type 1 error. Accordingly, the results of this study are exploratory, necessitating further future study with larger sample sizes. Finally, information is not available on the impact of the identified mutations on downstream gene and protein targets. Though we have been able to identify genes that are mutated in samples of DSRCT, further study is needed to decipher how these alterations affect downstream signaling pathways. With these limitations in mind, this study still contributes vital information to the genomic profile of DSRCT, identifying key mutations and pathognomonic features of the disease that can guide diagnosis, staging, and management in the future.

## 6. Conclusions

This study provides important information on both the demographic burden and genomic profile of DSRCT. Overall, the observed genomic mutation landscape is consistent with previously reported molecular profiles of DSRCT. However, disparities in diagnosis burden of the disease by demographics demonstrated greater representation of White patients compared to Non-White patients. These findings emphasize the importance of further studying EWSR1:WT1 and its downstream targets to identify new targets for DSRCT treatment. This will be invaluable for improving diagnostic ability, addressing poor prognosis, and guiding care and management of DSRCT.

## Figures and Tables

**Table 1:** Demographic Data for DSRCT.

Demographics	Category	n (%)
Sex	Male	117 (82.4%)
	Female	22 (15.5%)
	Unknown	3 (2.1%)
Age category	>18 years old	118 (58.4%)
	≤18 years old	84 (41.6%)
Ethnicity	Non-Hispanic	107 (75.4%)
	Unknown/Not Collected	9 (6.3%)
	Hispanic	23 (16.2%)
	Not collected	3 (2.1%)
Race	Asian	12 (8.5)
	White	84 (59.2)
	Black	27 (19%)
	Other	7 (4.9)
	Unknown	9 (6.3)
Sample Type	Primary	95 (47%)
	Metastasis	97 (48%)
	Other	10 (5.0%)

Table 1 highlights the demographic characteristics of included DSRCT patients queried using the AACR GENIE database. The value, “n (%),” represents the number and percentage of patients who fell into each demographic category.

**Table 2:** Frequency of Common Somatic Mutations in DSRCT.

Gene Mutation	n (%)
*ARID1A*	16 (7.9)
*TP53*	5 (2.5)
*ATM*	7 (3.5)
*TERT*	6 (3.0)
*FGFR4*	5 (2.5)
*EP300*	4 (2.0)
*ALK*	3 (1.5)
*NOTCH1*	4 (2.0)
*KMT2C*	4 (2.0)
*MTOR*	4 (2.0)

Table 2 highlights the frequency, depicted in the column entitled “n (%),” of commonly occurring somatic mutations seen in DSRCT.

**Table 3:** Frequency of Common CNAs in DSRCT.

	Gene	n (%)
Homozygous Deletion	*CRLF2*	7 (3.6)
	*PTEN*	5 (2.6)
	*FAT1*	5 (2.7)
	*CDKN2A*	4 (2.1)
	*CDKN2B*	4 (2.1)
Amplification	*TMPRSS2*	4 (2.1)
	*ERG*	3 (1.6)
	*ICOSLG*	3 (1.7)
	*U2AF1*	3 (1.6)
	*ELF3*	3 (2.0)

Table 3 demonstrates the most common homozygous deletion and amplification mutations in DSRCT. Values are indicated in the column entitled “n (%).”

**Table 4:** Common DSRCT mutations by sex.

Gene (Chi-Squared)	Male, n	Female, n	*p* Value
*FL1*	0	1	*p* = 5.78 × 10^−3^
*NSD1*	2	3	*p* = 0.0142
*RUNX1*	0	2	*p* = 0.0157
*STAG2*	1	2	*p* = 0.0434
*WT1*	145	17	*p* = 0.0489

Table 4 stratifies common mutations in DSRCT by sex. Statistical significance was determined using the threshold of *p* < 0.05.

**Table 5:** Common DSRCT mutations by race.

Gene (Chi-Squared)	Non-White, n	White, n	*p* Value
*FLI1*	1	0	*p* = 8.33 × 10^−3^
*KDM2B*	1	0	*p* = 0.0165
*MAGED1*	1	0	*p* = 0.0165
*MKI67*	1	0	*p* = 0.0165
*PCLO*	1	0	*p* = 0.0165
*TRAF5*	1	0	*p* = 0.0165
*AKAP9*	0	1	*p* = 0.0323
*ANKRD11*	3	0	*p* = 0.0344
*KMT2C*	4	1	*p* = 0.0411

Table 5 demonstrates common mutations seen in DRCT by race. Statistical significance was determined using the threshold of *p* < 0.05.

## Data Availability

The data presented in this study are available in the AACR GENIE database (v17.0), which can be found at the following link: https://genie.cbioportal.org/ (accessed 9 June 2025). Data that was extracted from this repository has been included in this manuscript and its corresponding tables.

## Abbreviations

The following abbreviations are used in this manuscript:

DSRCT	Desmoplastic Small Round Cell Tumor
AACR GENIE	American Association for Cancer Research Genomics Evidence Neoplasia Information Exchange
UICC	Union for International Cancer Control
CNA	Copy Number Alteration
